# A clinical trial of group-based body psychotherapy to improve bodily disturbances in post-treatment cancer patients in combination with randomized controlled smartphone-triggered bodily interventions (KPTK): study protocol

**DOI:** 10.1186/s40359-019-0357-1

**Published:** 2019-12-30

**Authors:** Astrid Grossert, Cornelia Meffert, Viviane Hess, Christoph Rochlitz, Miklos Pless, Sabina Hunziker, Brigitta Wössmer, Ulfried Geuter, Gunther Meinlschmidt, Rainer Schaefert

**Affiliations:** 1 Department of Psychosomatic Medicine, University Hospital Basel, Basel, Switzerland; 2 Department of Medical Oncology, University Hospital Basel, Basel, Switzerland; 3 Division of Clinical Psychology and Psychotherapy, Department of Psychology, University of Basel, Basel, Switzerland; 4 Faculty of Medicine, University of Basel, Basel, Switzerland; 5 Department of Medical Oncology, Winterthur Cantonal Hospital, Winterthur, Switzerland; 6 Outpatient practice for psychotherapy, Olten, Switzerland; 7 Institute for Sports and Motology, University of Marburg, Marburg, Germany; 8 Division of Clinical Psychology and Cognitive Behavioral Therapy, International Psychoanalytic University, Berlin, Germany; 9 Division of Clinical Psychology and Epidemiology, Department of Psychology, University of Basel, Basel, Switzerland

**Keywords:** Bodily disturbances, Body awareness, Body psychotherapy, Cancer, Group, Integrative body psychotherapy, Psycho-oncology, Quality of life, Smartphone-triggered interventions

## Abstract

**Background:**

Disturbances in bodily well-being represent one key source of suffering and impairment related to cancer. There is growing evidence that body psychotherapy (BPT) is efficacious for the treatment of various mental disorders. However, with regard to cancer patients, evidence is scarce. The aims of this project are to evaluate whether bodily disturbances in post-treatment cancer patients can be improved by group BPT, and to estimate the efficacy of intermittent smartphone-triggered bodily interventions.

**Methods:**

The project is a bi-center study with two participating centers in Switzerland, applying a pre-post convergent parallel design of a weekly group BPT using a waiting-period comparator, including a nested RCT during the group BPT phase. During the BPT phase, either a smartphone-triggered bodily intervention or a smartphone-triggered control intervention is provided at random over 5 consecutive weeks, on 6 days weekly. Patients who had received curatively intended treatment for any malignant neoplasm (treatment being completed ≥3 months) and are suffering from bodily disturbances are screened to assess eligibility. Sample size estimation is based on an a priori power analysis. We plan to include a total of *N* = 88 subjects, aiming at at least 52 completers.

Patients are surveyed three times (baseline assessment (T0), pre- (T1) and post-intervention assessment (T2)), and on a daily basis along BPT during five consecutive weeks. The primary outcome, bodily disturbances, is assessed using the ‘Body Image Scale‘(BIS). For the secondary outcomes standardized questionnaires are used to assess changes in experience of presence and vitality, mood, body mindfulness, somatic symptoms and somatic symptom disorder, quality of life, anxiety, and depression including suicidal tendency, vitality and mental health, as well as group cohesion. Using semi standardized interviews (at T0 and T2), we aim to explore the relation of BPT with bodily disturbances and body image in post-treatment cancer patients, as well as the acceptance and burden of the intervention.

**Discussion:**

The proposed study has strong potential benefits for cancer patients, as it may pave the way for new therapeutic approaches to treat bodily disturbances, which persist despite curative tumor therapy. These may considerably improve patients’ biopsychosocial well-being and quality of life.

**Trial registration:**

ClinicalTrials.gov NCT03707548 (registered 9 October 2018; retrospectively registered).

## Background

Cancer is a major public health issue and related to a high burden of disease. With an increasing number of patients surviving cancer, the high individual cancer-related burden is of growing importance. As recently indicated by the Global Burden of Disease (GBD), this burden is not only caused by fear of mortality but also by physical and psychosocial impairment [1–3]. It is not only caused by the tumor and its treatment but also originates from cancer-related experiences and the suffering caused by the disease. Notably, cancer related burden may persist even if the neoplasm has been treated successfully [4–8].

Cancer-related impairments often go along with disturbances in bodily well-being [9–14]. However, bodily disturbances are multidimensional, and varying and sometimes conflicting definitions are used [9, 15–17]. When using the term in the context of our study, we refer to the definition of Rhoten. She identified key aspects of body image disturbances which include the self-perception of change in appearance and displeasure with this change, a decline concerning various aspects of physical functioning and the psychological distress caused by these changes [16].. Body image disturbances are highly prevalent in cancer patients [9, 18, 19] and may persist despite successful interventions that target the tumor itself. They pose a major challenge to the well-being and quality of life of cancer patients and require to be appropriately addressed by care providers.

There is some evidence that body psychotherapy (BPT), defined as ‘psychotherapeutic treatment of mental disease or suffering, concomitantly using bodily and mental psychotherapeutic means’ (see [20]) is efficacious for the treatment of various mental disorders [21–24]. However, with regard to cancer patients, evidence of BPT is scarce [25, 26]. BPT explicitly targets bodily aspects, such as perceptions, feelings, and attitudes towards the body, which are of paramount importance in the context of bodily disturbances in cancer patients. Therefore, scrutinizing BPT as an intervention to reduce disturbances of bodily well-being appears to be highly promising. It may offer possibilities to directly aim at treatable mechanisms, which are the cause of cancer-related disturbances in bodily well-being. These reflections are in line with the recently suggested focus on an ‘experimental therapeutic approach’ of the National Institute of Mental Health as one of the most prominent funding institutions in this field [27].

Our study aims at evaluating the potential of body psychotherapy (BPT) to address cancer-related bodily disturbances. The thereby applied intervention *‘group body psychotherapy for post-treatment cancer patients’* is based on BPT as an experience-oriented approach [20, 28, 29]. The overall goal of this group BPT is to relieve bodily disturbances, caused or triggered by the preceding cancer and related treatments. Thus, the group BPT should support patients to learn how to cope with undesirable bodily sensations, feelings, and disturbances, such as changes in overt body image [30, 31] as well as changes in attitudes towards and perceptions of their own body [32]. This includes feelings of insecurity and vulnerability [33–35], of being stigmatized [10], of impaired functioning [35, 36], as well as feelings of disconnectedness from one’s own body [34].

### Study aims and objectives

The aims of this trial are to evaluate whether bodily disturbances in post-treatment cancer patients can be improved by group BPT, and to estimate the efficacy of intermittent smartphone-triggered bodily interventions (German acronym ‘KPTK: **K**örper**p**sycho**t**herapie bei **K**rebs’ in English: BPT for cancer patients. For trial registration data see Additional file 1). We assume that bodily disturbances will improve from pre- to post-BPT in post-treatment cancer patients. Furthermore, participants will show better immediate outcomes with regard to mood and bodily well-being if they receive smartphone-triggered bodily interventions as compared to smartphone-triggered control interventions. With this non-randomized evaluation of a weekly group BPT using a waiting-period comparator, with a nested randomized controlled trial (RCT) we will primarily obtain information on the efficacy of the intervention. Furthermore, we will be able to investigate intervention effects and mechanisms of action in more detail, together with acceptance and perception of the intervention, unwanted effects and burden to patients.

The primary objective of the planned project is to evaluate whether group BPT is related to reduction of bodily disturbances in post-treatment cancer patients.

Secondary objectives of the project are

1. to assess if BPT is related to more body awareness/mindfulness;
2. to estimate, whether intermittent smartphone-triggered bodily interventions go along with immediate improvements in bodily well-being
3. to evaluate, whether BPT is related to improvements in mental well-being (anxiety, depression, somatization, pain, etc.) and quality of life;
4. to assess group processes/cohesion;
5. to assess aspects of the intervention, such as
  - recruitment and inclusion;
  - undesired side effects (‘safety assessment’);
  - from the patients’ perspectives:

(i) acceptance and burden of the intervention (this assessment is recommended according to recent initiatives, such as ‘Partnering with Patients’ launched by the British Medical Journal (BMJ) [37]);
(ii) suggestions for improvement of the intervention.

## Methods/design

**Fig. 1:**
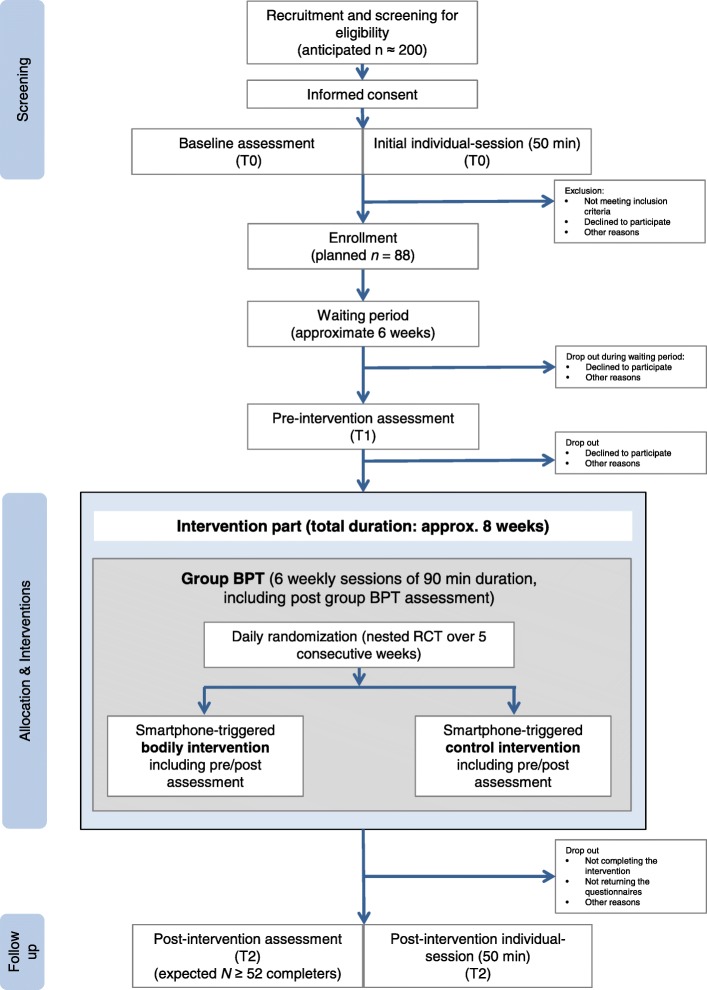
Outline of design and participant flow of the study

In this non-randomized evaluation of a weekly group BPT with a nested RCT (smartphone-triggered bodily interventions during group BPT) participants first undergo a waiting period (duration if possible 6 weeks; given that recruitment for a group intervention is difficult, reasonable exceptions of a shorter waiting period are allowed) followed by the group BPT (6 weekly sessions, 90 min each). During the group BPT, either a smartphone-triggered bodily intervention or a smartphone-triggered control intervention is provided at random (randomization on a daily basis) over a period of 5 consecutive weeks on 6 days per week. The project is based on a convergent parallel design. We apply quantitative and qualitative assessments, as described in more detail below. We depict the outline of the study design and the flow of study participants in Fig. 1.

Following recommendations from the BMJ to improve patient involvement in research [37] we involved two patients, who had participated in the first conducted BPT group [25] beyond the study descript here, in the translation process of the BIS and the development of semi-standardized questionnaires, as well as in the preparation of the study information. Furthermore, both were asked to report on their experiences, and to review the planned trial and the study materials regarding their practical applicability and acceptance. When reporting the study, we took into account the guidelines and recommendations of the Consolidated Standards of Reporting Trials (CONSORT) and Transparent Reporting of Evaluations with Non-randomized Designs (TREND) statements [38, 39] and we adhered the guidelines for Standard Protocol Items: Recommendations for Interventional Trials (SPIRIT), see Additional file 2 [40].

### Study sample

We intend to include a total of 88 patients (44 at each study side). With an expected response rate of approximately 70%, we aim at a sample size of 52 completers (26 per participating center). The therapeutic intervention provided in this study aims at post-treatment cancer patients suffering from disturbances in bodily well-being. Patients having received curatively intended treatment for malignant neoplasms at the participating institutions, are screened according to the eligibility and exclusion criteria outlined in Table 1.

**Table 1:** Inclusion and exclusion criteria

*Inclusion criteria:*	
• Having received curatively intended treatment for any malignant neoplasm;	
• Primary treatment (surgery, radiotherapy, chemotherapy) being completed ≥3 months ago before study inclusion. Any other ongoing anti-tumor therapy is allowed (e.g., hormonal therapy, adjuvant immunotherapy);	
• Bodily disturbances (BIS ≥10) (= clinical cutoff for body image dissatisfaction [9]); OR ((BIS = 2–9) AND ((patient-assessed distress due to bodily changes (VAS-B; 0–10) ≥ 5) OR (therapist-assessed awareness of bodily changes (VAS-A) ≥ 5 AND therapist-assessed related distress due to bodily changes (VAS-B, 0–10) ≥ 5)))	
• No sign of progress or recurrence of malignancy at study inclusion according to treating physician;	
• Score of 0 or 1 according to the PS of the ECOG [41];	
• Having an anticipated life expectancy of ≥12 months, according to treating physician (recent evidence suggests that this is the best source for prediction of survival [42]);	
• Age 18 years or older;	
• Capacity to participate in group BPT sessions in Basel or Winterthur, 3 study assessments, and the smartphone-triggered interventions;	
• Ability to provide informed consent.	
*Exclusion criteria:*	
• Suffering from a severe current mental disorder;	
• Risk of current suicidality, as indicated by a suicide item score ≥ 2 in the BDI-II [43], as this group BPT intervention is not appropriate to support suicidal patients in acute crises. (Given a score ≥ 2, need for support will be assessed and if required, patients are instructed to call for or transferred to local psychiatric support);	
• Participation in any other clinical trial with a psychosocial intervention;	
• Receiving any other current psychotherapeutic treatment with the exception of already existing long-lasting therapies (≥ 6 months);	
• Inability to understand and speak German.	

*Abbreviations*: *BDI* Beck Depression Inventory, *BPT* Body psychotherapy, *ECOG* Eastern Cooperative Oncology Group, *PS* Performance score, *VAS* Visual analog scale

### Recruitment and screening

Recruitment takes place at University Hospital Basel and at the Cantonal Hospital Winterthur. Further, potential participants are approached via public advertisements (e.g. advertisement in public transport and on the website of the Basel Cancer League). Patients having received curatively intended treatment for any malignant neoplasm (treatment being completed ≥3 months) and suffering from bodily disturbances due to the cancer diagnosis and treatment are provided with oral and written information about the study and asked if they are willing to participate. Patients are informed by trained research nurses or the project leader. In addition, the date and time of the specific BPT group are clarified with each participant in advance. Once written informed consent has been obtained, patients are screened with baseline assessment (T0), including standardized questionnaires and a semi-structured baseline interview whether they are eligible for the BPT intervention or not. Non-eligible patients are provided with information regarding alternative therapeutic support. Included patients undergo a waiting period of 6 weeks (given that recruitment for a group intervention is difficult, reasonable exceptions of a shorter waiting period are allowed) followed by the pre-intervention assessment (T1), weekly assessments after each group BPT session, and daily pre−/post smartphone-triggered assessments. After completion of the group BPT phase, the post-intervention assessment (T2) with standardized questionnaires and a semi-structured post-intervention interview takes place (Table 3 gives an overview of assessment instruments and time points).

### Withdrawal and discontinuation

Study participation is voluntary and can be withdrawn at any time during the study. If patients withdraw consent to participate in the study due to of any personal reasons, they will not be excluded from participation in group BPT. An investigator may terminate participation in the study if any clinical adverse events or medical situations occur and the continued participation in the study would not be in the best interest of the participant. Participation may also be terminated if the participant meets an exclusion criterion (either newly developed or previously not recognized) that does not allow further study participation. Thus, according to the “Withdrawal of Subjects from Research Guidance” [47], already collected data that is related to any participant who chooses to withdraw from the study will be retained and analyzed. We will anonymize these data after data evaluation has been completed.

### Risk-benefit assessment

The study provides body-psychotherapeutic support for cancer patients. Essentially, we expect neither risks nor additional burdens to patients. However, in psychotherapy unwanted adverse effects might occur. Often it is difficult to distinguish between negative life events, undesirable developments of the disease, and side effects of psychotherapy [48]. Exposure to one’s own bodily disturbances could lead to increased short-term physical and mental distress. Nonetheless, we expect that potential distress can be dealt with directly during the intervention, given that trained, experienced psychotherapists conduct the BPT.

### Interventions

#### Group BPT intervention

The first author developed the intervention based on integrative body psychotherapy approaches [20, 28, 29], adapted to cancer patients and their needs and opted for group setting. Compared to individual therapy, group interventions may benefit from additional therapeutic factors and may have economic benefits [49, 50]. First experiences with group BPT for cancer patients were obtained from an initial group (6 patients), as described elsewhere [25]. The intervention is carried out in small groups within 6 sessions, 90 min each. The intended time frame for conducting all 6 sessions is 6–8 weeks (public holidays etc. included). The group BPT is provided as part of the outpatient service of University Hospital Basel and Cantonal Hospital Winterthur, using facilities from the Cancer Leagues Basel and Zürich in close proximity to the hospitals.

The 6 group BPT sessions will comprise the following topics: 1) general introduction, fostering of group cohesion and focus on bodily perception; 2) focus on bodily resources and grounding; 3) focus on closeness and distance regulation; 4) focus on social interactions and bodily impulses; 5) focus on embodied emotions; and 6) summary and transfer session. All sessions should proceed along the following phases: A) Opening: brief bodily exercise and exchange, preparing the specific topic of the session; B) Psycho-educational element and exercises triggering embodied experiences, focusing on the specific topic of the session with sharing (reflection and exchange of experiences during the exercise); C) Closure: résumé and farewell (see Table 2 and Grossert et al. [25]). Within this schedule, each session can be adapted to the composition of the current patient group and its respective needs. Thus, group processes can be addressed accordingly. In between sessions, patients are instructed to continue the exercises (supported by smartphone-based triggers, see below), making sure that tools, experiences, and strategies are transferred and integrated into their daily lives. To improve intervention adherence, participants are contacted, if they do not attend a group appointment without having given prior notice. Patients are informed that they can contact us at any time if they have any uncertainties or questions.

**Table 2:** Content of interventions: Group body psychotherapy with cancer patients and smartphone based bodily interventions

	Content group body psychotherapy				Content audiofiles
Session	Topic	Opening including short feedback on the previous session	Introduction & Exercise including sharing 1) Introduction and Psychoeducation 2) Strategies/Exercise 3) Sharing of own experiences	Closure including perspective for the upcoming week	Smartphone based bodily interventions supporting transfer from group BPT sessions into daily live
Duration	In Total 90 min	15–20 min	50–65 min	10–15 min	randomly on 3 days a week 10–15 min
1.	Group cohesion and body perception and awareness	▪ Self-introduction ▪ Expectations and fears	1) - Reflection about bodily perception, body image, body disturbances and body work experience- Introduction of BPT terms 2) - Breath perception - Body awareness with BodyScan technique, supine position- Self - contact (hands on/off) 3) Reflection about own experience during the exercises	What can I take with me after this first contact in the group?	Body awareness with body scan technique
2.	Bodily resources and grounding/ anchoring	▪ Short body scan CEB ▪ Breath perception ▪ Feedback	1) - Body as a resource - Balance between distress and resources, bodily stress reaction, adapted [44] 2) - Body awareness with BodyScan technique, standing position - Foot work and anchoring with the focus on connection to stability, e.g. p97 [28]- Movement perception including mirroring 3) Reflection about own experience during the exercises	How to transfer exercise skills into daily life?	Anchoring/grounding with footwork using a small rubber ball
3.	Boundary awareness	▪ Short body scan CEB ▪ Anchoring exercise ▪ Feedback	1) Space and boundaries including importance of having the choice between own and shared space 2) Boundary awareness: - Lika Breathing technique, p37 [45] - Boundary awareness: exploring own space and own boundaries, p84 [28] 3) Reflection about own experience during the exercises	Transfer of boundary awareness into daily life experience.	Boundary awareness through Lika breathing technique
4.	Impulses and social/group interaction	▪ Short body scan CEB ▪ Lika breathing technique, p37 [45] ▪ Feedback	1) Social interactions and (body) impulses to get into/out of social interactions 2) – Body self-release techniques, adapted p209 [28] - Awareness of bodily perception and the nature of impulses - nonverbal contact Interaction with different body parts 3) Reflection about own experience during the exercises	How can I find a witness position being aware of perception and impulses? What do I need?	Relaxation through body self-release techniques
5.	Embodied emotions	▪ Short body scan CEB ▪ Anchoring exercise ▪ Feedback	1) Integration model of human experience, p26 [28] 2) - Mapping of feelings under the cancer disease and treatment - Focus on embodiment: body sculptures of emotions, adapted [46] 3) Reflection about own experience during the exercises	How to become aware of feelings and how to explore and share them in daily life?	Self-awareness through ‘four body zone’ exercise
6.	Summary	▪ Short body scan CEB ▪ Free choice of exercise repetition ▪ Feedback	1) Summary and open questions 2) Free choice of exercise repetition 3) Closing: Ritual “*being connected while continue on individual path*”	What would I take with me? What would I leave in this group/room? Evaluation	

*BPT* Body Psychotherapy, *CEB* Cognition, emotions, body perception

Group BPT is provided by three trained psychotherapists. In order to guarantee continuity within each group, one single therapist leads all 6 sessions of a specific group. Therapists have a professional background in terms of either a medical or psychological degree, followed by specialized training in integrative body psychotherapy (IBP; accredited by the Federation of Swiss Psychologists). Furthermore, they then receive training in the ‘group BPT for post-treatment cancer patients’ approach by the first author of the present study according to the specific manual which describes the group content in detail (manual not yet published, for an outline see Table 2.). At the beginning and end of completing a series of the six BPT session, mandatory peer consulting of the therapy is provided by the first author and then continuously ensured according to the needs of the group leader. In case the first author is conducting the group, continuous supervision of the therapy is provided by a senior body psychotherapist.

#### Smartphone-triggered interventions

The smartphone-triggered bodily interventions consist of brief BPT exercises aiming at supporting the transfer from the group BPT sessions into patients’ daily lives. Smartphone bodily interventions are triggered by short audio-clips, as described elsewhere [51]. The patients are asked to log into the system each day once. Then, they randomly receive either an audio-clip triggering a bodily intervention (3 times a week) or a control intervention (3 times a week). In case of technical difficulties, participants can contact the study team. The content of the bodily interventions is outlined in Table 2. The control interventions consist of 15 selected fairy tales all adapted approximately to the same length as the bodily interventions. The advantage of fairy tales is their universality and distance to the content of cancer diagnosis or its treatment. The smartphone-triggered bodily and control interventions are provided over a period of 5 consecutive weeks on 6 days per week, in parallel to the BPT sessions. Thus, each patient undergoes 15 bodily and 15 control interventions.

### Assignment of smartphone-triggered interventions

An independent party (Clinical Trial Unit (CTU) of the University Hospital Basel) generated the computer-generated random sequences, using the software R, allowing individual randomization of every training day of each trial participant to the bodily or control interventions (within-subject randomization). Randomization was blocked every six training days for each trial participant to ensure that during each training week, each subject is triggered for three bodily and three control interventions of the pre-specified 6 weekly exercises per trial participant over the 5 consecutive weeks. No further restrictions applied. The series of random sequences generated by CTU was provided to a collaborator, who sequentially linked each patient after enrolment with the next sequence on the list. Trial participants were blinded to randomization up until the moment at which the intervention was provided; Body psychotherapists (care providers) were blinded to randomization. Outcome assessment on each smartphone-based intervention day was conducted directly via smartphone, so outcome assessor blinding is not applicable.

### Assessments

For all assessments, we apply validated instruments with good quality criteria. Sociodemographic variables are assessed at baseline (T0) only. All other constructs are assessed three times: at baseline after study enrollment (baseline assessment; T0), after the waiting period (pre-intervention assessment; T1), and after completion of the group BPT (post-intervention assessment; T2).

Within the group intervention, bodily disturbances, body mindfulness and group cohesion is evaluated weekly after each group BPT session. Experience of presence and experience of vitality and mood are additionally assessed pre- and post-smartphone-triggered interventions. Furthermore, therapist’s adherence to the manual is recorded with a respective checklist adapted to the session’s context. Table 3 gives an overview of outcome measures, assessment instruments, and time points.

**Table 3:** Outcome measures, assessment instruments, and assessment time points

Measures	Assessment instruments	Baseline Assessment T0	Pre-Intervention Assessment T1	Weekly assessments after group BPT	Pre/Post smartphone-triggered intervention	Post-Intervention Assessment T2
Social demographics	Sociodemographic variables (sex, age, marital status, educational level, and job-related situation)	X				
Psychosocial health	Basic Documentation for Psycho-Oncology (PO-Bado)	X				
Performance status	Performance status score of the Eastern Cooperative Oncology Group (ECOG)	X				
Bodily disturbances	Body Image Scale (BIS)	X	X	X		X
Awareness of bodily changes due to the diagnosis and treatment	single item; VAS-A patient-assessed (range 0–10)	X	X			X
Distress caused by bodily changes	single item; VAS-B patient-assessed (range 0–10)	X	X			X
Body mindfulness	Body Mindfulness Questionnaire (BMQ)	X	X	X		X
Distress	National Comprehensive Cancer Network Distress Thermometer (DT)	X	X		X	X
Somatic Symptoms	Somatic Symptom Disorder-B Criteria Scale (SSD-12)	X	X			X
Quality of life	EORTC QLQ-C30	X	X			X
Vitality	36-Item Short Form Health Survey (SF-36) – Scale Vitality	X	X			X
Mental health	36-Item Short Form Health Survey (SF-36) – Scale Mental Health	X	X			X
Anxiety and depression	Hospital Anxiety and Depression Scale (HADS)	X	X			X
Suicidal tendency	Beck Depression Inventory (BDI-II), Item I: Suicidal tendency	X	X			X
Group cohesion	Group Climate Questionnaire – Short Form (GCQ-S)			X		X
Mood	Multidimensional Mood Questionnaire (MDMQ)				X	
Experience of presence	single item; VAS (range 0–10)				X	
Experience of vitality	single item; VAS (range 0–10)				X	
Therapist’s rating of patient’s bodily changes due to the diagnosis and treatment	Semi-standardized Interview single item; VAS-A therapist-assessed (range 0–10)	X				
Therapist’s rating of patient’s distress caused by bodily changes	Semi-standardized Interview single item; VAS-B therapist-assessed (range, 0–10)	X				
Motivation, previous experience, eligibility	Semi-standardized Interview	X				
Acceptance, treatment effect, potential for improvement, safety aspects	Semi-standardized Interview					X
Therapist’s adherence to the manual	Adapted checklist to session content (see Table 2.)			X		

*BPT* Body Psychotherapy, *EORTC QLQ-C30* European Organization for Research and Treatment of Cancer QLQ-C30, *VAS* Visual Analog Scale

The primary outcome, bodily disturbances, is assessed using the ‘Body Image Scale‘(BIS), which is a brief 10-item scale validated in cancer patients, showing sensitivity to change and high reliability (Cronbach’s alpha 0.93) and validity [52]. We translated this questionnaire from English into German according to the European Social Survey Translation Guidelines [53].

Secondary outcomes are assessed using the ‘Body Mindfulness Questionnaire’ (BMQ) with high internal consistencies (Cronbach’s alpha = 0.93 for “Experiencing Body Awareness” and 0.91 for “Appreciating Body Awareness”) [54], the ‘Somatic Symptom Disorder-B Criteria Scale‘(SSD-12) with high reliability (Cronbach’s alpha = 0.95) [55], the ‘Hospital Anxiety and Depression Scale’ (HADS, Cronbach’s alpha = 0.93 for “Anxiety” and 0.90 for “Depression”) [56, 57], and the ‘Multidimensional Mood Questionnaire‘(MDMQ; Cronbach’s alpha = 0.92) [58, 59]. Quality of life is assessed using the ‘European Organization for Research and Treatment of Cancer’ (EORTC QLQ-C30) questionnaire [60] with good reliability (Cronbach’s alpha = 0.82 for “Physical functioning”; 0.90 for “Role functioning”, 0.84 for “Emotional functioning”, 0.72 for “Cognitive functioning”, 0.86 for “Social functioning”, 0.86 for “Global Quality of Life, 0.84 for “Fatique”, 0.58 for “Nausea/vomiting”, and 0.86 for “Pain” [61]. And two scales (Vitality and Mental Health) of the ‘Short Form Health Survey ’ (SF-36, Cronbach’s alpha of 0.86 for Vitality and 0.84 for Mental Health) [62]. Additional information is collected using the ‘Basic Documentation for Psycho-Oncology’ (PO-Bado) [63], the ‘National Comprehensive Cancer Network Distress Thermometer‘(DT) [64, 65], and via the assessment of the performance status score of the Eastern Cooperative Oncology Group (ECOG) [66]. We apply single item VAS (0–10) to assess experience of presence and experience of vitality. The ‘Group Climate Questionnaire – Short Form‘(GCQ-S) [67] is used to assess group climate.

The baseline assessment includes a semi-standardized individual face-to-face interview (30–50 min). During this interview the group therapist addresses the most relevant key-issues regarding the intervention, as well as previous experiences, expectations, and concerns. Finally a semi-standardized individual face-to-face interview is conducted by the group therapist, to address treatment effect, effect mechanism, acceptance, and potential of improvement and safety aspects, as well as whether they would recommend participation in the intervention to other patients. The need for further psychological support is clarified in the final interview. If further psychological support is required, contact information of respective providers will be provided. The semi-structured interviews are audiotaped if participants provide respective informed consent. Afterwards, they are transcribed and evaluated according to Mayring [68] in order to investigate acceptance, treatment effects and mechanism, burden, and potential for improvement of the interventions. Exclusions, recruitment, and dropout rates will be recorded.

### Sample size estimation

Sample size of the planned project is based on an a priori power analysis. With 52 participants completing the group BPT (study site Basel: *n* = 26, study site Winterthur: *n* = 26), we will have sufficient power (1-*β* = 0.94) to gain pre-post differences of medium effect size (*d* = 0.5) in the primary outcome. Allowing for a 30% dropout rate and including a safety margin of 10% accounting for unexpected variation in our estimates, we aim to include a total of *N* = 88 patients. With regard to the nested RCT evaluating the short-term efficacy of smartphone-triggered bodily interventions, power analysis is more demanding. Given a maximum of 15 bodily interventions and 15 control interventions per person and assuming a participation rate in daily interventions of 80% (which is a rather conservative estimate, as compared to the previously observed 96% participation rate in an earlier study [51]), we expect on average a total of 24 completed smartphone-triggered interventions per participant. Assuming a correlation of 0.5 among repeated measures and a nonsphericity correction ε of 1, we expect sufficient power (1-*β* > 0.99) to detect medium effect sizes (*f* = 0.25) (estimation conducted using G*Power 3.1 [69], based on an analysis of variance (ANOVA) model with repeated measures and within factors).

### Statistical and content analyses

Descriptive analyses of continuous variables will include the calculation of central tendency and dispersion; descriptive analyses of categorical variables will include numbers and frequencies.

We intend to use inferential statistics to compare outcomes and parameters over time: (1) from baseline to post group BPT intervention and (2) from pre to post smartphone-triggered bodily intervention. We intend to compare changes from T1 to T2 with changes from T0 to T1. Therefore, we intend to use mixed effect generalized linear models, which will also be used to assess potential mediators of therapeutic changes. Baseline equivalence between T0 and T1 will be assessed in order to adjust potential baseline differences and that way to reduce potential bias arising from the nonrandomized study design. We intend to conduct additional analyses, including adjusted (e.g., age, gender) and subgroup analyses (e.g., cancer entity, BPT group composition, level of somatic distress). All statistical tests will be two-sided, and *p*-values ≤0.05 will be considered statistically significant. In the event of multiple comparisons, Bonferroni-Holm corrections will be carried out.

We intend to analyze qualitative data based on thematic analyses, aiming at identifying themes within the data and at establishing meaningful categories, their interrelation, and their relation to the outcomes of the intervention [68, 70].

Intention-to-treat analyses will include all patients being enrolled in the study and randomized. Per protocol analyses will follow an all-patients-receiving-intervention perspective. Whenever appropriate, we are going to use multiple imputation methods [71].

### Safety aspects and monitoring

If an adverse event occurs. The project leader is promptly notified if immediate safety and protective measures have to be taken during the conduct of the research project. The Ethics Committee will be notified of these measures and of the circumstances necessitating them within 7 days. If a serious adverse event occurs, the research project will be interrupted and the Ethics Committee notified on the circumstances within 7 days according to HRO Art. 21.An independent party monitors the study (Category A according to ordinance HRO Art.7).

## Discussion

The treatment of cancer patients is a major challenge and often relies on administering medication such as cytotoxic agents [7]. In most cases these treatments go along with physical and psychological distress and additional approaches are required to improve health related quality of life in cancer patients. In recent years, psychosocial interventions have gained increasing importance [72–75]. Therapies such as cognitive behavioral therapy (CBT) that have proven to be effective in non-cancer contexts often show little or no effects in cancer patients [76–79]. Additionally, studies assessing complementary interventions, such as music-based interventions or mindfulness-based stress reduction (MBSR), showed beneficial effects in cancer patients; yet results were often heterogeneous [80–84]. There is growing evidence that movement-based interventions for cancer patients (in terms of physical activity and exercise) are safe and feasible and improve quality of life. Effect sizes, however, are mostly small [85–91]. Some studies have applied body-related interventions in cancer patients, such as Tai Chi, acupressure, or Qi-Gong. Within a larger intervention program they were, however, of minor importance, which makes it difficult to draw any conclusions regarding the effects of these bodily interventions [92–94].

Furthermore, some of the above-mentioned studies which included movement-based interventions have successfully incorporated home-based exercises in addition to face-to-face interventions [93, 95]. Apart from being used in cancer treatment, those psychotherapeutic interventions have shown positive effects. Recent evidence indicates that the implementation of new mobile technologies, such as smartphones, may help to increase therapeutic efficacy, when applied within a blended psychotherapy approach [96–99]. However, to the best of our knowledge, smartphone-triggered bodily interventions have not yet been applied and assessed in the context of cancer on a daily basis over five consecutive weeks.

Overall, the proposed study has strong potential benefit for cancer patients, as it may pave the way for new therapeutic approaches to treat bodily disturbances, which persist despite tumor therapy. Through a better coping with the experience of bodily sensations and disturbances cross-linked with the emotional and cognitive experience, patients’ biopsychosocial well-being and quality of life might be considerably improved.

In conclusion, the intervention evaluated in this study has the potential to be of high scientific and social value, as it will provide the basis for more differentiated and evidence-based interventions to support cancer patients, regarding persistent bodily disturbances. This may not only lead to reduced suffering and impairment, but may also result in outcomes, such as better family functioning, social functioning, etc.. The intervention may be expected to be cost-efficient, due to its conceptualization as group therapy. It will contribute to our understanding of the applicability of BPT to physical illnesses in general, and beyond mental disorders. Moreover it will allow a better understanding whether and how new technologies can be successfully combined with classic therapeutic face-to-face settings (‘blended psychotherapy’). Further, it will improve our understanding of therapeutic mechanisms of BPT in cancer patients. It will provide all necessary information to conduct a subsequent international phase III RCT on the topic. Finally, this project will contribute to enhance interdisciplinary and integrative cancer research and will further support the growing number of cancer survivors from a more comprehensive perspective.

## Supplementary information

**Additional file 1.** Trial registration data.

**Additional file 2.** Spirit Checklist.

## Data Availability

With this manuscript we provide the study protocol but no patient data.
