# Supplementary material for: A clinical trial of group-based body psychotherapy to improve bodily disturbances in post-treatment cancer patients in combination with randomized controlled smartphone-triggered bodily interventions (KPTK): study protocol
Source: BMC Psychol. 2019 Dec 30;7:90. doi: 10.1186/s40359-019-0357-1 (PMC6936033; doi:10.1186/s40359-019-0357-1)
Supplement: Supplementary file 1 — Additional file 1. Trial registration data. [file 40359_2019_357_MOESM1_ESM.docx]

Additional file 1. *Trial registration data*

| Data category | Information |
| --- | --- |
| Primary registry and trial identifying number | ClinicalTrials.gov NCT03707548 |
| Secondary identifying numbers | 2018-01115; me17Schaefert2 |
| Dates of registration in primary registry | - First submitted: September 26, 2018 - First Submitted that Met Quality Control Criteria consistent with National Library of Medicine (NLM): October 9, 2018 - First posted: October 16, 2018 - Last Update Posted : June 12, 2019 |
| Source(s) monetary or material support | Swiss Cancer League / Swiss Cancer Research Grant-No: KLS-4304-08-2017 University Hospital Basel, Switzerland |
| Sponsor | Prof. Dr. Rainer Schaefert, Department of Psychosomatics, Division of Medicine, University Hospital Basel, Switzerland |
| Contact for public queries | RS, AG |
| Contact for scientific queries | RS, GM, AG |
| Public title | KPTK = **K**örper**p**sychotherapie bei *K*rebs  In English: Bodypsychotherapy (BPT) for cancer patients |
| Scientific title | A clinical trial of group-based body psychotherapy to improve bodily disturbances in post-treatment cancer patients in combination with randomized controlled smartphone-triggered bodily interventions (KPTK):  study protocol |
| Countries of recruitment | Switzerland |
| Health condition(s) or problem(s) | Cancer patient, bodily disturbances |
| Interventions/treatment | **Experimental: Group BPT**  Six group BPT sessions (using a waiting-period comparator and pre-/post design)  **A nested randomized controlled trial (RCT)** is included  - Behavioral: Smartphone triggered bodily interventions  - Behavioral: Smartphone triggered control intervention |
| Key Inclusion criteria | - Having received curatively intended treatment for any malignant neoplasm; primary treatment (surgery, radiotherapy, chemotherapy) being completed >/= 3 months ago before study inclusion. Any other ongoing anti-tumor therapy is allowed (e.g., hormonal therapy, adjuvant immunotherapy)  - Existing bodily disturbances |
| Ages eligible for study | 18 years or older |
| Inclusion and exclusion criteria | See Table 1. Inclusion and exclusion criteria |
| Study type | Interventional |
| Allocation | Study design: The project follows the outline of a non-randomized evaluation of a weekly group BPT using a waiting-period comparator, with a nested randomized controlled trial (RCT) to evaluate the short-term efficacy of smartphone-triggered bodily interventions |
| Primary purpose | Treatment |
| Date of first enrolment | September 3, 2018 |
| Target sample size | 88 participants |
| Recruitment status | Active, not recruiting |
| Primary outcome(s) | Changes in bodily disturbances |
| Key secondary outcomes | Changes in bodily wellbeing and mood  Changes in body mindfulness, somatic symptoms/somatic symptom disorder, distress, anxiety & depression, mental health and quality of life |
