# Supplementary material for: A clinical trial of group-based body psychotherapy to improve bodily disturbances in post-treatment cancer patients in combination with randomized controlled smartphone-triggered bodily interventions (KPTK): study protocol
Source: BMC Psychol. 2019 Dec 30;7:90. doi: 10.1186/s40359-019-0357-1 (PMC6936033; doi:10.1186/s40359-019-0357-1)
Supplement: Supplementary file 2 — Additional file 2. Spirit Checklist. [file 40359_2019_357_MOESM2_ESM.doc]

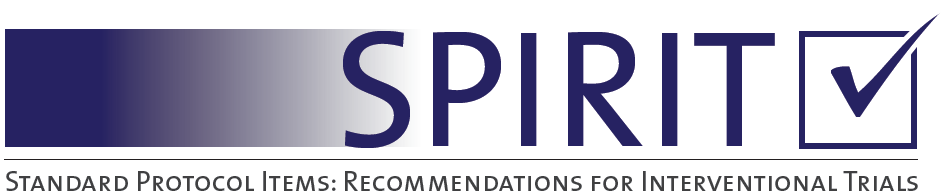


SPIRIT 2013 Checklist: Recommended items to address in a clinical trial protocol and related documents*

| Page/section | Section/item | ItemNo | | Description | |
| --- | --- | --- | --- | --- | --- |
| Grossert et al. A clinical trial of group-based body psychotherapy to improve bodily disturbances in post-treatment cancer patients in combination with randomized controlled smartphone-triggered bodily interventions (KPTK): study protocol | **Administrative information** | | | | |
| p1, title | Title | 1 | | Descriptive title identifying the study design, population, interventions, and, if applicable, trial acronym | |
| p3 | Trial registration | 2a | | Trial identifier and registry name. If not yet registered, name of intended registry | |
| p3, provided as *additional file Trial registration data* |  | 2b | | All items from the World Health Organization Trial Registration Data Set | |
| p10, section *Ethics approval, consent to participate, and monitoring* Version 3.0, dated 26.01.2019 | Protocol version | 3 | | Date and version identifier | |
| P10, section *funding* | Funding | 4 | | Sources and types of financial, material, and other support | |
| p1 names, affiliation  p10, section *authors’ contributions* | Roles and responsibilities | 5a | | Names, affiliations, and roles of protocol contributors | |
| p1, corresponding author |  | 5b | | Name and contact information for the trial sponsor | |
| p10, section *authors’ contributions* and *funding* |  | 5c | | Role of study sponsor and funders, if any, in study design; collection, management, analysis, and interpretation of data; writing of the report; and the decision to submit the report for publication, including whether they will have ultimate authority over any of these activities | |
| p10, section *authors’ contributions*  and *staff list* provided |  | 5d | | Composition, roles, and responsibilities of the coordinating centre, steering committee, endpoint adjudication committee, data management team, and other individuals or groups overseeing the trial, if applicable (see Item 21a for data monitoring committee) | |
|  | Introduction |  | |  | |
| P2, section *background*, paragraph study aims and objectives  p5, section r*isk-benefit assessment* | Background and rationale | 6a | | Description of research question and justification for undertaking the trial, including summary of relevant studies (published and unpublished) examining benefits and harms for each intervention | |
| p7, section s*martphone-triggered interventions* |  | 6b | | Explanation for choice of comparators | |
| P3, section study aims and objectives | Objectives | 7 | | Specific objectives or hypotheses | |
| p2, abstract  p3, section *methods/design* | Trial design | 8 | | Description of trial design including type of trial (eg, parallel group, crossover, factorial, single group), allocation ratio, and framework (eg, superiority, equivalence, noninferiority, exploratory) | |
|  | Methods: Participants, interventions, and outcomes | | | | |
| P3, section *recruitment and screening* | Study setting | 9 | | Description of study settings (eg, community clinic, academic hospital) and list of countries where data will be collected. Reference to where list of study sites can be obtained | |
| P5, Table 1. *Inclusion and exclusion criteria* | Eligibility criteria | 10 | | Inclusion and exclusion criteria for participants. If applicable, eligibility criteria for study centres and individuals who will perform the interventions (eg, surgeons, psychotherapists) | |
| p5, section *group Body psychotherapy intervention*  p7; section *smartphone triggered interventions*  p6 Table 2. *Content of interventions: Group body psychotherapy with cancer patients and smartphone based bodily interventions* | Interventions | 11a | | Interventions for each group with sufficient detail to allow replication, including how and when they will be administered | |
| p5, section *withdrawal* and *discontinuation* and section *risk-benefit assessment* and  p9 *Safety aspects and monitoring* |  | 11b | | Criteria for discontinuing or modifying allocated interventions for a given trial participant (eg, drug dose change in response to harms, participant request, or improving/worsening disease) | |
| P3, section *recruitment and screening*  p5, section *group Body psychotherapy intervention*  p7, section s*martphone-triggered interventions* |  | 11c | | Strategies to improve adherence to intervention protocols, and any procedures for monitoring adherence (eg, drug tablet return, laboratory tests) | |
| P5, Table 1. *Inclusion and exclusion criteria* |  | 11d | | Relevant concomitant care and interventions that are permitted or prohibited during the trial | |
| p2, section *background*  p3, section *study aims and objectives*  p5, section *risk-*  *benefit assessment*  p7, section *assessment*  p8, Table 3*. Outcome measures, assessment instruments, and assessment time points* | Outcomes | 12 | | Primary, secondary, and other outcomes, including the specific measurement variable (eg, systolic blood pressure), analysis metric (eg, change from baseline, final value, time to event), method of aggregation (eg, median, proportion), and time point for each outcome. Explanation of the clinical relevance of chosen efficacy and harm outcomes is strongly recommended | |
| P4, Figure 1. *Outline of design and participant flow of the study* | Participant timeline | 13 | | Time schedule of enrolment, interventions (including any run-ins and washouts), assessments, and visits for participants. A schematic diagram is highly recommended (see Figure) | |
| P9, section s*ample size estimation* | Sample size | 14 | | Estimated number of participants needed to achieve study objectives and how it was determined, including clinical and statistical assumptions supporting any sample size calculations | |
| P3, section *recruitment and screening* | Recruitment | 15 | | Strategies for achieving adequate participant enrolment to reach target sample size | |
|  | **Methods: Assignment of interventions (for controlled trials)** | | | | |
|  | Allocation: |  | |  | |
| p7, section *smartphone-triggered interventions*    Stratification factors: NA  🡪 *Note: Document describing randomisation plan in detail was sent as additional file to markrannie.manangan@springernature.com* | Sequence generation | 16a | | Method of generating the allocation sequence (eg, computer-generated random numbers), and list of any factors for stratification. To reduce predictability of a random sequence, details of any planned restriction (eg, blocking) should be provided in a separate document that is unavailable to those who enrol participants or assign interventions | |
| p7, section *assignment of smartphone-triggered interventions* | Allocation concealment mechanism | 16b | | Mechanism of implementing the allocation sequence (eg, central telephone; sequentially numbered, opaque, sealed envelopes), describing any steps to conceal the sequence until interventions are assigned | |
| p7, section *assignment of smartphone-triggered interventions* | Implementation | 16c | | Who will generate the allocation sequence, who will enrol participants, and who will assign participants to interventions | |
| p7, section assignment of smartphone-triggered interventions | Blinding (masking) | 17a | | Who will be blinded after assignment to interventions (eg, trial participants, care providers, outcome assessors, data analysts), and how | |
| NA |  | 17b | | If blinded, circumstances under which unblinding is permissible, and procedure for revealing a participant’s allocated intervention during the trial | |
|  | **Methods: Data collection, management, and analysis** | | | | |
| p7, section *assessment*  p29, Table 3*. Outcome measures, assessment instruments, and assessment time points* | Data collection methods | 18a | | Plans for assessment and collection of outcome, baseline, and other trial data, including any related processes to promote data quality (eg, duplicate measurements, training of assessors) and a description of study instruments (eg, questionnaires, laboratory tests) along with their reliability and validity, if known. Reference to where data collection forms can be found, if not in the protocol | |
| p5, section *withdrawal and discontinuation*  list of any outcome data: NA |  | 18b | | Plans to promote participant retention and complete follow-up, including list of any outcome data to be collected for participants who discontinue or deviate from intervention protocols | |
| Details of data management are described in the protocol Version 3.0, dated 26.1.2019, section 7 *quality control and data protection, p25*. | Data management | 19 | | Plans for data entry, coding, security, and storage, including any related processes to promote data quality (eg, double data entry; range checks for data values). Reference to where details of data management procedures can be found, if not in the protocol | |
| P9, section *statistical and content analyses* | Statistical methods | 20a | | Statistical methods for analysing primary and secondary outcomes. Reference to where other details of the statistical analysis plan can be found, if not in the protocol | |
| p9, section *statistical and content analyses* |  | 20b | | Methods for any additional analyses (eg, subgroup and adjusted analyses) | |
| p9, section *statistical and content analyses* |  | 20c | | Definition of analysis population relating to protocol non-adherence (eg, as randomised analysis), and any statistical methods to handle missing data (eg, multiple imputation) | |
|  | **Methods: Monitoring** | | | | |
| NA  due to minimal risks --> study categorization: Category A acc. To ordinance HRO Art.7 (see study protocol V3.0, dated 26.1.2019) | Data monitoring | 21a | | Composition of data monitoring committee (DMC); summary of its role and reporting structure; statement of whether it is independent from the sponsor and competing interests; and reference to where further details about its charter can be found, if not in the protocol. Alternatively, an explanation of why a DMC is not needed | |
| NA |  | 21b | | Description of any interim analyses and stopping guidelines, including who will have access to these interim results and make the final decision to terminate the trial | |
| p9, section s*afety aspects and monitoring* | Harms | 22 | | Plans for collecting, assessing, reporting, and managing solicited and spontaneously reported adverse events and other unintended effects of trial interventions or trial conduct | |
| p9, section s*afety aspects and monitoring*  Please note according to study categorization: Category A acc. To ordinance HRO Art.7, no monitoring is recommended, even so we implemented a monitoring plan. | Auditing | 23 | | Frequency and procedures for auditing trial conduct, if any, and whether the process will be independent from investigators and the sponsor | |
|  | Ethics and dissemination | | | | |
| p10, section *ethics approval and consent to participate* | Research ethics approval | 24 | | Plans for seeking research ethics committee/institutional review board (REC/IRB) approval | |
| p10, section *ethics approval and consent to participate*  *🡪 respective, additional information of all ethics approvals were send to markrannie.manangan@springernature.com* | Protocol amendments | 25 | | Plans for communicating important protocol modifications (eg, changes to eligibility criteria, outcomes, analyses) to relevant parties (eg, investigators, REC/IRBs, trial participants, trial registries, journals, regulators) | |
| P3, section *recruitment and screening* | Consent or assent | 26a | | Who will obtain informed consent or assent from potential trial participants or authorised surrogates, and how (see Item 32) | |
| p7, section *assessments*,  additional consent: informed consent for audiotaping interview |  | 26b | | Additional consent provisions for collection and use of participant data and biological specimens in ancillary studies, if applicable | |
| section *confidentiality and coding*  Not mentioned in the study protocol manuscript: --> please note: *Project data will be handled with utmost discretion and will only be accessible to authorized persons and stored securely at the study site and secured server. On the CRFs and other project specific documents, participants are only identified by unique participant number. All data of the participants are stored with the participant number, which does not allow inference on their identity. The assignment of patient names to participant numbers will be documented in a patient list that will be kept under lock. Data will be stored for at least ten years*. | Confidentiality | 27 | | How personal information about potential and enrolled participants will be collected, shared, and maintained in order to protect confidentiality before, during, and after the trial | |
| p10, section *competing interests* | Declaration of interests | 28 | | Financial and other competing interests for principal investigators for the overall trial and each study site | |
| Not mentioned in the study protocol manuscript:  Please note 🡪 *Investigators of the project will have access to data. The clinical study agreement between Sponsor and second study site regulates publication rights. This document is proven by the ethics committee*. | Access to data | 29 | | Statement of who will have access to the final trial dataset, and disclosure of contractual agreements that limit such access for investigators | |
| P7, section *assessment* | Ancillary and post-trial care | 30 | | Provisions, if any, for ancillary and post-trial care, and for compensation to those who suffer harm from trial participation | |
| Not mentioned in the study protocol manuscript:  Please note🡪 *we intent to publish results in journals and conferences without any publication restrictions.*  *At this time, there is no data sharing arrangement planned* | Dissemination policy | 31a | | Plans for investigators and sponsor to communicate trial results to participants, healthcare professionals, the public, and other relevant groups (eg, via publication, reporting in results databases, or other data sharing arrangements), including any publication restrictions | |
| Not mentioned in the study protocol manuscript:  *The authorship eligibility guidelines of the International Committee of Medical Journal Editors (ICMJE) have been observed.*  *There was no use of professional writers.* |  | 31b | | Authorship eligibility guidelines and any intended use of professional writers | |
| NA, not planned |  | | 31c | | Plans, if any, for granting public access to the full protocol, participant-level dataset, and statistical code |
|  | Appendices |  | |  | |
| Informed consents for study participating and audiotaping, and patient information are proven by the ethics committee. | Informed consent materials | 32 | | Model consent form and other related documentation given to participants and authorised surrogates | |
| NA | Biological specimens | 33 | | Plans for collection, laboratory evaluation, and storage of biological specimens for genetic or molecular analysis in the current trial and for future use in ancillary studies, if applicable | |

*It is strongly recommended that this checklist be read in conjunction with the SPIRIT 2013 Explanation & Elaboration for important clarification on the items. The SPIRIT checklist is copyrighted by the SPIRIT Group under the Creative Commons “[Attribution-NonCommercial-NoDerivs 3.0 Unported](http://www.creativecommons.org/licenses/by-nc-nd/3.0/)” license.

V1.0; Basel, 9^th^ of August 2019
